# The role of the laparoscopic approach in the surgical management of acute adhesive small bowel obstruction

**DOI:** 10.1186/s12893-019-0504-x

**Published:** 2019-04-24

**Authors:** Enric Sebastian-Valverde, Ignasi Poves, Estela Membrilla-Fernández, María José Pons-Fragero, Luís Grande

**Affiliations:** 1 Department of Surgery, Hospital del Mar, Universitat Autònoma de Barcelona, Passeig Marítim 25-29, 08003 Barcelona, Spain; 2 IMIM (Hospital del Mar Medical Research Institute), Barcelona, Spain

**Keywords:** Tissue adhesions, Small intestine, Intestinal obstruction, Laparoscopy, Length of stay

## Abstract

**Background:**

Postoperative adhesions represent 75% of all acute small bowel obstructions. Although open surgery is considered the standard approach for adhesiolysis, laparoscopic approach is gaining popularity.

**Methods:**

A retrospective study with data from a prospectively maintained data base of all patients undergoing surgical treatment for adhesive small bowel obstruction (ASBO) from January 2007 to May 2016 was conducted. Postoperative outcomes comparing open vs laparoscopic approaches were analysed. An intention to treat analysis was performed. The aim of the study was to evaluate the potential benefits of the laparoscopic approach in the treatment of ASBO.

**Results:**

262 patients undergoing surgery for ASBO were included. 184 (70%) and 78 (30%) patients were operated by open and laparoscopic approach respectively. The conversion rate was 38.5%. Patients in the laparoscopic group were younger (*p* < 0.001), had fewer previous abdominal operations (*p* = 0.001), lower ASA grade (*p* < 0.001), and less complex adhesions were found (p = 0.001). Operative time was longer in the open group (*p* = 0.004). Laparoscopic adhesiolysis was associated with a lower overall complication rate (43% vs 67.9%, *p* < 0.001), lower mortality (*p* = 0.026), earlier oral intake (p < 0.001) and shorter hospital stay (p < 0.001). Specific analysis of patients with single band and/or internal hernia who did not need bowel resection, also demonstrated fewer complications, earlier oral intake and shorter length of stay. In the multivariate analysis, the open approach was an independent risk factor for overall complications compared to the laparoscopic approach (Odds Ratio = 2.89; 95% CI 1.1–7.6; *p* = 0.033).

**Conclusions:**

Laparoscopic management of ASBO is feasible, effective and safe. The laparoscopic approach improves postoperative outcomes and functional recovery, and should be considered in patients in whom simple band adhesions are suspected. Patient selection is the strongest key factor for having success.

## Background

Postoperative adhesions are the most common cause of acute small bowel obstruction, representing 75% of all cases. Around 50% will require surgical treatment, laparotomy being the preferred approach [1]. The morbidity and mortality of adhesiolysis remain significant, with rates around 14–45 and 4% respectively [2–6]. The laparoscopic approach has demonstrated benefits in other urgent and elective situations, offering lower morbidity, less postoperative pain and shorter hospital stay, even in adhesive small bowel obstruction (ASBO) [1, 7]. Nevertheless, laparotomy continues to be considered the standard surgical approach at most centres [1].

The main drawbacks related with the laparoscopic approach in ASBO are: risk of intraoperative bowel injury, difficulty handling the bowel loops, difficulty obtaining a correct view of the cause of the obstruction and the presumably higher cost of the procedure [8]. However, in the guides of the World Society of Emergency Surgery Adhesive Small Bowel Obstruction working group [9, 10], only factors related to pneumoperitoneum (hemodynamic instability or cardiopulmonary impairment) are considered absolute exclusion criteria for laparoscopic approach. The absence of prospective randomized studies and the lack of consensus in the indications regarding the most appropriated approach have meant that laparoscopy remains the second option in the surgical treatment of ASBO.

The aim of our study is to evaluate the impact of the laparoscopic approach on postoperative outcomes in our series of patients consecutively operated for ASBO. We hypothesize that the laparoscopic approach is a feasible choice for the ASBO and achieves better outcomes than the open approach in selected patients.

## Methods

After Institutional Review Board approval, a retrospective study was conducted using data from a prospectively maintained data base of the patients who underwent laparoscopic or open surgery for ASBO (including internal hernias) at our centre from January 2007 to May 2016. Only urgent operations were considered for the analysis. Following the protocol of our centre, when an ASBO is suspected, a nasogastric tube is placed. If the suspected diagnosis of adhesions is supported by clinical and radiological data, once the stomach is empty, 100 ml of water soluble oral contrast is administered in those patients with previous abdominal operations. Those in whom oral contrast did not reach the colon in 24 h were considered as a failure of the conservative management and surgical intervention is indicated. Patients in whom the cause of the obstruction was not ASBO (primary or secondary neoplasm, gallstone ileus, bezoar, post-radiotherapy stenosis), were excluded, as well as obstructions caused by any type of abdominal wall hernia.

The variables analysed were: a) preoperative: age, sex, American Society of Anesthesiologists (ASA) score, number of previous abdominal operations, previous abdominal mesh; b) intraoperative: surgeon’s experience in advanced laparoscopy, surgical approach, conversion, operative time, intraoperative findings, intraoperative injury; c) postoperative: morbidity, mortality, onset of oral intake, reoperation, length of hospital stay, readmissions and quality outcomes. The postoperative complications were assessed according to the Clavien-Dindo classification [11].

Simple adhesion was defined as a single well-defined band adhesion, clearly causing the bowel obstruction. Otherwise, adhesions were classified as complex. Expertise in advanced laparoscopy was considered for those surgeons with experience in more than 50 gastrointestinal, bariatric, colorectal or advanced hepato-bilio-pancreatic laparoscopic procedures and experience in intracorporeal suturing.

Any assisted incision or laparotomy was considered to be a conversion regardless of its length. Postoperative complications were recorded during hospitalization and up to 30 days after discharge. Mortality was assessed in-hospital or at 30-day postoperative. Quality outcomes were measured using the Poor Quality Outcomes (PQO) variable. PQO was considered when a patient presented: a major complication (Clavien-Dindo IIIb-V), or a minor complication (II and IIIa) but with a prolonged hospital stay beyond 15 days, and/or readmission within 30 days of discharge.

The surgical approach of choice was decided by individual surgeons according to their personal criteria and laparoscopic skills. Hemodynamic instability, suspicion of intestinal ischemia, clearly hostile abdomen and patients with medical contraindications for pneumoperitoneum were considered contraindications for the laparoscopic approach.

### Statistical analysis

Both the Chi-Square test and the Fisher’s exact test were used for categorical variables when appropriate, and the Mann-Whitney U test for continuous variables which did not follow a normal distribution. An intention-to-treat analysis was performed in which all the conversions were included in the laparoscopic group. The significant variables in the bivariate analysis were included in the multivariate analysis with a logistic regression. Results are expressed as n (%), mean values ± standard deviation (SD) or as median (interquartile range (IQR)). A *p* value less than 0.05 was considered significant. The standard program of the Statistical Package for the Social Sciences (IBM® SPSS® Statistics Version 20, Chicago, IL, USA) was used for all the statistical analysis.

## Results

A total of 262 patients underwent surgical operation for ASBO, 78 (29.8%) by laparoscopy and 184 (70.2%) by open approach. Table 1 shows the patient’s baseline preoperative characteristics of the entire sample and for the subgroups. Patients who underwent laparotomy were significantly older, had more previous abdominal operations and a higher ASA grade.

**Table 1:** Patient’s baseline demographics and summary of preoperative data

	Total *N* = 262	Laparoscopy *n* = 78	Open *n* = 184	p
Age	66.06 ± 18.7	59.36 ± 18.7	68.9 ± 18	< 0.001
Female gender	137 (52.3)	41 (52.6)	96 (52.2)	0.954
Previous abdominal operation	225 (85.9)	62 (79.5)	163 (88.6)	0.053
N°. of previous abdominal operations	1.87 ± 1.6	1.4 ± 1.2	2.07 ± 1.7	0.001
Previous wall mesh placement	69 (26.5)	15 (19.5)	54 (29.5)	0.095
ASA				< 0.001
I	22 (8.7)	13 (17.1)	9 (5.1)	
II	97 (38.2)	41 (53.9)	56 (31.5)	
III	112 (44.1)	20 (26.3)	92 (51.7)	
IV	23 (9.1)	2 (2.6)	21 (11.8)	

Values are n (%) unless otherwise specified as mean (± SD)

Table 2 displays the intraoperative and postoperative variables. Although about 50% of all the ASBO were caused by complex adhesions, single bands and/or internal hernias predominated in the laparoscopy group and complex bands in the open group. 21% of the patients required intestinal resection, which was more frequent in open group (*p* = 0.014). However, the adhesion type was not associated with the need of intestinal resection (*p* = 0.743). 21 of 262 patients required a reoperation. In Table 3, are shown the causes of reoperation and the deaths for both laparoscopic and open groups.

**Table 2:** Perioperative data, complications and postoperative variables

	Total N = 262	Laparoscopy n = 78	Open n = 184	p
Advanced laparoscopic skills	70 (26.8)	46 (59)	24 (13.1)	< 0.001
Intraoperative findings				0.001
Single band or internal hernia	129 (49.2)	53 (67.9)	76 (41.3)	
Complex adhesions	133 (50.8)	25 (32.1)	108 (58.7)	
Operative time	120.75 ± 60.5	103.11 ± 48.2	128.41 ± 63.8	0.004
Intraoperative injury	28 (10.7)	6 (7.7)	22 (12)	0.307
Postoperative complications				< 0.001
No	103 (39.3)	44 (56.4)	59 (32.1)	
Yes	159 (60.7)	34 (43.6)	125 (67.9)	
Clavien-Dindo				
I-II-IIIa	113 (43.1)	28 (35.9)	85 (46.2)	0.102
IIIb-IV-V	46 (17.6)	6 (7.7)	40 (21.7)	
Mortality	17 (6.5)	1 (1.3)	16 (8.7)	0.026
Reoperation	21 (8)	5 (6.4)	16 (8.7)	0.533
Poor Quality Outcomes	86 (33.1)	13 (16.7)	73 (40.1)	< 0.001
Onset of oral intake	5.18 ± 5.4	3.83 ± 4.1	5.85 ± 5.8	< 0.001
Length of hospital stay	9 (5–15)	5 (3–10)	11 (7–17)	< 0.001
30-day postoperative readmissions	17 (7)	3 (3.9)	14 (8.4)	0.201

Values are n (%) unless otherwise specified as mean ± SD or median (IQR)

**Table 3:** Causes of reoperation in laparoscopic and open group

Causes	ITT Laparoscopy *n* = 5	Open *n* = 16
Haemorrhage	1 (^a^)(^b^)	2 (^a^) (^c^)
Evisceration	1	4 (^a^)
Anastomotic leak	–	3 (^a^)
Missed bowel injury	1	2
Intraabdominal abscess	1	2
Surgical Wound Infection	–	1
Early adhesions recurrence	1	2

*ITT* intention to treat

(^a^) Death of a patient

(^b^) Haemorrhage of the epigastric artery due to a paracentesis in a cirrhotic patient

(^c^)One subcutaneous bleeding(^a^) and one bladder haemorrhage in patient with a iatrogenic ureter injury

Thirty patients (38.5%) were converted due to: technical difficulty in 20; need for extensive bowel resection in five; intestinal intraoperative injury in four; and trocar haemorrhage in one. Comparison of outcomes between non-converted and converted patients of the laparoscopic group is shown in Table 4. No differences were found in age, ASA or number of previous surgeries. Significant differences were found in the conversion rate among the experts and non-experts in laparoscopic approach (26.1% vs 56.1%; *p* = 0.007). but this was not associated to fewer reoperations (8.7% vs 3.1%; *p* = 0.643) nor fewer complications (41.3 vs 46.9%; *p* = 0.626) respectively.

**Table 4:** Comparison of perioperative data, complications and postoperative variables between non-converted and converted patients in laparoscopic group

	Non-converted *n* = 48	Converted *n* = 30	p
Advanced laparoscopic skills	34 (70.8)	12 (40)	0.007
Intraoperative findings			0.029
Single band or internal hernia	37 (77.1)	16 (53.3)	
Complex adhesions	11 (22.9)	14 (46.7)	
Intestinal resection	2 (4.2)	7 (23.3)	0.023
Operative time	78 ± 30.7	143.8 ± 43.6	< 0.001
Intraoperative injury	1 (2.1)	5 (16.7)	0.029
Postoperative complications	9 (18.8)	25 (83.3)	< 0.001
Mortality	0 (0)	1 (3.3)	0.385
Reoperation	1 (2.1)	4 (13.3)	0.069
Poor Quality Outcomes	4 (8.3)	9 (30)	0.012
Onset of oral intake	2.2 ± 2.7	6.3 ± 4.6	< 0.001
Length of hospital stay	4 (1–7)	10 (1–19)	< 0.001

Values are n (%) unless otherwise specified as mean ± SD or median (IQR)

Seventeen (6.5%) patients died. Although no significant differences were found, mortality rate was higher in patients with complex adhesions (9% vs 3,9%; *p* = 0.091) and bowel resection (10.9% vs 5.3%; *p* = 0.213). The mean age of the 17 patients who died was 80.5 years, 8 were ASA IV, 12 had complex adhesions and 6 required intestinal resection.

Table 5 shows Odds Ratio (OR) for each variable for overall complications in the bivariate and multivariate analyses. The open approach presented an OR = 2.74 (95% CI 1.59–4.72; *p* < 0.001) in the bivariate analysis, however, in the multivariate analysis it did not reach statistical significance (OR = 1.58; 95% CI 0.78–3.22; *p* = 0.204), due to an increase in complications in converted patients (OR = 21.66; 95% CI 6.5–72.15; p < 0.001). In the multivariate analysis, age, the need for intestinal resection and the presence of complex adhesions were associated with an increase in complications.

**Table 5:** Bivariate and logistic regression analysis for overall complications

	BIVARIATE		MULTIVARIATE Laparoscopy vs Open	
	OR (95% CI)	p	OR (95% CI)	p
Approach				
Laparoscopy	1		1	
Open	2.74 (1.59–4.72)	< 0.001	1.58 (0.78–3.22)	0.204
Totally laparoscopic	1			
Open	9.18 (4.17–20.19)	< 0.001		
Converted	21.66 (6.5–72.15)	< 0.001		
Age	1.03 (1.02–1.05)	< 0.001	1.03 (1–1.04)	0.002
Male gender	0.94 (0.57–1.55)	0.828	0.99 (0.56–1.74)	0.987
N°. of previous abdominal operations	1.32 (1.09–1.61)	0.005	1.13 (0.92–1.38)	0.237
ASA	2.17 (1.52–3.1)	< 0.001	1.25 (0.81–1.92)	0.311
Intestinal resection	2.48 (1.26–4.9)	0.009	2.2 (1–4.7)	0.044
Complex adhesions	2.54 (1.52–4.23)	< 0.001	2 (1.1–3.6)	0.021
Advanced laparoscopic skills	0.7 (0.4–1.22)	0.212	1.18 (0.58–2.41)	0.647

On Table 6 are shown the results of the subgroup of patients operated for single bands and/or internal hernias without intestinal resection. Compared to open approach in this subgroup, laparoscopy presented significant lower morbidity, less PQO, earlier oral intake and shorter length of hospital stay. In the multivariate anlaysis (Table 7), the open approach emerged as an independent factor for the increase in complications. On contrary, among the patients with complex adhesions and/or those patients who required intestinal resection, we did not find differences in reoperation (12.9% vs 8.6%; *p* = 0.495), complications (67.7% vs 72.7%; *p* = 0.586) and PQO (29% vs 44.1%; *p* = 0.127), between laparoscopic and open group respectively. There were also no differences in operative time (*p* = 0.926), oral intake (*p* = 0.371) and hospital stay (*p* = 0.079).

**Table 6:** Patient demographics, perioperative and postoperative data for patients with single band or internal hernias without intestinal resection

	Total *n* = 103	Laparoscopy *n* = 47	Open *n* = 56	p
Age	64.6 ± 18.8	60.43 ± 18	68.11 ± 18.8	0.021
Female gender	57 (55.3)	26 (55.3)	31 (55.4)	0.997
Previous abdominal operations	83 (80.6)	36 (76.6)	47 (83.9)	0.349
N°. of previous abdominal operations	1.47 ± 1.17	1.34 ± 1.2	1.57 ± 1.1	0.187
ASA				0.098
I	11 (10.8)	6 (13)	5 (8.9)	
II	51 (50)	28 (60.9)	23 (41.1)	
III	35 (34.3)	11 (23.9)	24 (42.9)	
IV	5 (4.9)	1 (2.2)	4 (7.1)	
Operative time	90.7 ± 46.3	81.93 ± 38	98.17 ± 51.45	0.188
Intraoperative injury	2 (1.9)	1 (2.1)	1 (1.8)	1
Postoperative complications				0.003
No	58 (56.3)	34 (72.3)	24 (42.9)	
Yes	45 (43.7)	13 (27.7)	32 (57.1)	
Clavien-Dindo				0.134
IIIa	33 (32)	12 (25.6)	21 (37.5)	
IIIb-IV-V	12 (11.7)	1 (2.1)	11 (19.6)	
Mortality	2 (1.9)	0 (0)	2 (3.6)	0.191
Reoperation	6 (5.8)	1 (2.1)	5 (8.9)	0.216
Poor Quality Outcomes	21 (20.4)	4 (8.5)	17 (30.4)	0.006
Onset of oral intake	3.76 ± 3.2	2.39 ± 1.9	4.89 ± 3.6	< 0.001
Length of hospital stay	6 (4–11.5)	4 (3–8)	9.5 (6–15.25)	< 0.001

Values are n (%) unless otherwise specified as mean ± SD or median (IQR)

**Table 7:** Multiple regression analysis of single band or internal hernia without intestinal resection for overall complications

	MULTIVARIATE Laparoscopy vs Open	
	OR (95% CI)	p
Approach		
Laparoscopy	1	
Open	2.89 (1.1–7.6)	0.033
Age	1.03 (1–1.1)	0.034
Male gender	0.9 (0.4–2.2)	0.827
N°. of previous abdominal operations	1.07 (0.7–1.5)	0.734
ASA	1.6 (0.8–3.3)	0.203
Advanced laparoscopic skills	1.28 (0.5–3.6)	0.631

## Discussion

The results of our study show that the laparoscopic approach in the management of ASBO is associated with better postoperative outcomes, lower morbidity, fewer PQO, an earlier onset of oral intake and a shorter length of hospital stay, especially for selected patients with simple adhesions. The number and type of previous operations and peritoneal damage have been considered an important risk factors involved in the pathogenesis of adherences [12]. Some studies have also associated the size of the laparotomy with the formation of new adhesions [13], and have quantified the prevalence of postoperative adherences as high as 93% [14]. A study comparing two groups of 205 patients undergoing either laparoscopic and open colorectal surgery, did not find differences in admissions for intestinal obstruction (9% vs 13%) but reported a higher indication for surgery in the open approach when present (2% vs 8%; *p* = 0,006) [15]. Similarly, in a review by Burns et al. of 187,148 patients who underwent colorectal surgery, 3.5% required adhesiolysis within three years of surgery. In that study the patients who underwent laparoscopic approach, had a lower percentage of readmissions and less need for reoperation for adhesions (OR = 0.8; *p* < 0.001) [16]; in agreement with other studies [17, 18]. Therefore, laparotomy in the management of ASBO is in itself, a factor for the development of new episodes of ASBO and does not seem theoretically the best option.

Since Bastug reported the first laparoscopic adhesiolysis in 1991 [19], laparoscopic approach has demonstrated its feasibility and safety. Postoperative morbidity varies from 4 to 40% according to the series [20]. In a systematic review of 13,728 patients, Wiggins et al. [7], found a reduction in overall morbidity after laparoscopy (OR = 0.34; *p* = 0.0001) and other authors have corroborated these results [3, 4, 7, 21, 22]. In our study, we found a reduction in overall morbidity from 67.9 to 43.6% in the laparoscopic group. Although both rates are higher than those previously reported, we stress that 82.4% of the complications in laparoscopic group and 63.2% of those in the open group were minor (Clavien-Dindo I-II). Reoperation rates were 6.4 and 8.7% for laparoscopic and open approach respectively, which were both similar to those reported in other studies [7]. The Clavien-Dindo classification may underestimate or overestimate complications, regardless of the number of complications or their effect on length of hospital stay. For this reason, in the PQO variable we jointly analysed, the major complications, prolonged hospital stay and readmissions in order to assess which group presented worse outcomes overall. The laparoscopic approach showed better postoperative outcomes, so it seems that not only the number and grade of complications matters, but also in case of presenting complications, these seems to have less clinical impact.

One of the drawbacks of the laparoscopic approach is the possibility of intraoperative intestinal tearing during handling, especially in severe adhesions and multiple previous operations [9]. In a review of 19 studies including 1061 cases of ASBO operated by laparoscopy, rates of recognized intraoperative enterotomy and missed perforation were 6.5 and 0.8% respectively [6]. Dindo et al. [20] reported an intraoperative lesion rate of 9.5% and O’Connor et al. a rate of 6.6% [2]. Unlike most published studies, our study found a lower rate of perforation in laparoscopy, possibly due to the higher percentage of complex adhesions in open surgery (58.7% vs 32.1%) or to the low threshold for open conversion recommended by the guidelines [9]. Our results suggest that laparoscopy is a safe technique that does not increase the intraoperative risk of enterotomy.

A previous review of over 2000 cases reported a conversion rate as high as 36, and 6.7% of cases were considered laparoscopic-assisted [2]. A swiss registry also reported a conversion rate of 32.4% in 537 patients, including elective surgeries [20], and Ming-Zhe et al. published rates ranging from 26 to 51.9% [4]. In our study, any incision enlargement was considered a conversion, and so our conversion rate of 38.5%, is within the published limits. Some studies suggest that laparoscopic success depends on: early treatment (< 24 h after admission in emergency room), diameter of the bowel loops < 4 cm, a maximum of two previous surgeries, no previous midline laparotomies, single band adhesions, and the surgeon’s experience [9, 23]. The surgeon’s experience in advanced laparoscopy was a decisive factor in conversion in our study. One of our highlights, which has already been reported, is the increased morbidity and mortality related to conversion [5, 20, 24]. In our study, the complication rate was 83.3% in converted patients, although 80% were mild complications. This could be explained by a higher rate of complex adhesions and intestinal resection in converted group.

Since most conversions are due to technical difficulties and the inability to identify the cause of the obstruction, it is logical to think that patients with single adhesions and/or internal hernias without need for resection will be the ideal candidates for laparoscopy. The conversion rate for patients with simple adhesions fell to 23.4%. Moreover, in our study, the laparoscopy has not demonstrated to improve the outcomes in patients with complex and/or with intestinal resection. For this reason, further specific studies are now needed to determine the risk factors related to a higher probability to identify single adhesions and/or internal hernias; and to analyse risk factors for conversion. Our study has a selection bias already present in other similar retrospective studies [25], since patients in the laparoscopic group are younger, with a lower ASA score and fewer previous operations. This bias may alter postoperative outcomes compared to the open approach, and so prospective randomized studies are needed to validate the results obtained in this study. At present, only one randomized prospective trial is underway, scheduled to finish in 2018 [26].

Despite these limitations, we think that the results obtained are robust enough to confirm the benefits of the laparoscopic approach in ASBO, especially in selected patients with simple adhesions and when performed by surgeons skilled in advanced laparoscopic surgery.

## Conclusions

The laparoscopic approach for adhesive small bowel obstruction and/or internal hernias was associated with better postoperative outcomes, earlier oral intake, better quality outcomes and shorter length of hospital stay than the open approach, especially in selected patients with simple band adhesions. Patient selection is the strongest key factor for having success.

## Abbreviations

ASA: American Society of Anesthesiologists
ASBO: adhesive small bowel obstruction
IQR: interquartile range
OR: Odds Ratio
PQO: poor quality outcomes
